# The three-component helicase/primase complex of herpes simplex virus-1

**DOI:** 10.1098/rsob.210011

**Published:** 2021-06-09

**Authors:** Oya Bermek, R. Scott Williams

**Affiliations:** Genome Integrity and Structural Biology Laboratory, Department of Health and Human Services, National Institute of Environmental Health Sciences, National Institutes of Health, Research Triangle Park, NC 27709, USA

**Keywords:** herpesvirus DNA replication, helicase, primase, anti-viral drug

## Abstract

Herpes simplex virus type 1 (HSV-1) is one of the nine herpesviruses that infect humans. HSV-1 encodes seven proteins to replicate its genome in the hijacked human cell. Among these are the herpes virus DNA helicase and primase that are essential components of its replication machinery. In the HSV-1 replisome, the helicase–primase complex is composed of three components including UL5 (helicase), UL52 (primase) and UL8 (non-catalytic subunit). UL5 and UL52 subunits are functionally interdependent, and the UL8 component is required for the coordination of UL5 and UL52 activities proceeding in opposite directions with respect to the viral replication fork. Anti-viral compounds currently under development target the functions of UL5 and UL52. Here, we review the structural and functional properties of the UL5/UL8/UL52 complex and highlight the gaps in knowledge to be filled to facilitate molecular characterization of the structure and function of the helicase–primase complex for development of alternative anti-viral treatments.

## Graphical abstract

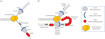

## 1. Introduction

Herpesviridae is a large family of approximately 100 double-stranded (ds) DNA viruses including nine that infect humans [1]. Herpesviruses are subdivided into alpha (*α*), beta (*β*) and gamma (*γ*) herpesvirinae [2]. The three subfamilies differ in the extent of genetic similarities, site of infection and length of cytolytic productive cycle [3]. A common feature among all herpesviruses is that following primary infection, the virus establishes lifelong latency in the infected person.

Herpesvirus infections are a complex threat to human health. In immunocompetent individuals, lytic (productive) herpesvirus infections result in an array of clinical presentations ranging from cold sores, genital lesions and chickenpox to mononucleosis and roseola (table 1). Moreover, in individuals with suppressed immune systems including neonates and those with diabetes, AIDS or cancer, opportunistic herpesvirus infections can cause life-threatening conditions. Herpes simplex virus 1 and 2 (HSV-1 and HSV-2) infections increase the risk of HIV infection [4,5]. In pregnant women, human cytomegalovirus (HCMV) can cause significant morbidity to fetuses and neonates [6]. Reactivation of latent human herpesvirus 6 and 7 (HHV-6 and HHV-7) infection, common after solid organ transplantation, has been associated with bone marrow suppression, encephalitis and pneumonitis [7]. Epstein–Barr virus (EBV) increases the risk of Hodgkin's lymphoma [8]. Kaposi's sarcoma caused by Kaposi's sarcoma-associated herpesvirus (KSHV) is the most common cancer in untreated AIDS patients [9]. The broad spectrum of illness linked to herpesvirus infections necessitates further molecular characterization of the herpes virus life cycle and the development of novel therapeutics.

**Table 1.** Herpesviridae infections. Abbreviations: Alpha (alphaherpesvirinae): HSV-1 (herpes simplex virus type 1), HSV-2 (herpes simplex virus type 2), VZV (varicella zoster virus); Beta (betaherpesvirinae): HCMV (human cytomegalovirus), HHV-6A (human herpesvirus 6A), HHV-6B (human herpesvirus 6B), HHV-7 (human herpesvirus 7); Gamma (gammaherpesvirinae): EBV (epstein–Barr virus), KSHV (Kaposi's sarcoma-associated herpesvirus).

subfamily	virus	symptoms in ommunocompetent hosts	symptoms in immunocompromised hosts
Alpha	HSV-1	herpes labialis, gingivostomatitis, keratoconjunctivitis, cutaneous herpes, genital herpes, encephalitis, viral meningitis	oesophagitis, pneumonia, disseminated infection, hepatitis
	HSV-2	genital herpes, cutaneous herpes, gingivostomatitis, neonatal herpes, viral meningitis	disseminated infection, hepatitis, encephalitis
	VZV	varicella (chickenpox), herpes zoster (shingles), viral meningitis	disseminated herpes zoster
Beta	HCMV	mononucleosis-like illness, hepatitis, cytomegalic inclusion disease, carditis	encephalitis, hepatitis, retinitis, pneumonia, colitis, gastrointestinal diseases, central nervous system diseases, pancreatitis, nephritis, allograft infections, oesophagitis
	HHV-6	roseola infantum, otitis media with fever	encephalitis, interstitial pneumonitis, partial myelosuppression, delayed engraftment, high-grade graft-versus-host-disease
	HHV-7	roseola infantum	encephalitis
Gamma	EBV	infectious mononucleosis, hepatitis, encephalitis, nasopharyngeal carcinoma, Burkitt's lymphoma, Hodgkin's lymphoma	lymphoproliferative syndromes, oral hairy leukoplakia, non-Hodgkin lymphoma
	KHSV		Kaposi's sarcoma, AIDS-related non-Hodgkin lymphoma, primary effusion lymphoma, multicentric Castleman disease, inflammatory cytokine syndrome

Most of the current drugs against herpesviruses target the virus-encoded DNA polymerase. These agents have been successfully used to treat HSV infections for decades, albeit less efficiently against varicella zoster herpesvirus (VZV) [10]. However, drug resistance developed by HSV poses a major problem for their effectiveness [11]. By contrast to the safety of HSV drugs, ganciclovir used for HCMV treatment has cytotoxic effects [12]. There are no therapies for HHV-6, HHV-7, EBV and KHSV. Herpesvirus infections remain a serious threat to human health, underscoring the need for characterization of the molecular mechanisms of herpes virus replication, with the goal of developing novel treatments for these widespread diseases.

The linear, double-stranded HSV-1 genome (152 kb) encodes as many as 284 open reading frames [13] of which seven gene products are dedicated to replicate its own genome in the infected human cell [14–16]. The essential gene products include the origin-binding protein (UL9), the core replisome composed of the single-strand DNA-binding protein (ICP8), *UL30* (DNA polymerase) and *UL42* (processivity factor) genes, and the heterotrimeric primosome encoded by the *UL5* (helicase), *UL52* (primase) and *UL8* (non-catalytic subunit) genes (table 2). These genes are all located within the long unique (U_L_) region of the genome, and they were named according to their genomic locations [14]. The primary amino acid sequence of six core genes is conserved among all animal and human herpesviruses. The high-level amino acid sequence conservation observed for the replication machinery implies that all human herpesviruses share the same lytic replication mechanism.

**Table 2.** Herpesviridae replication proteins in all nine human herpesviruses. NCBI's reference sequence (RefSeq) accession numbers of HSV-1 replication proteins: UL30 (YP_009137105.1), UL42 (YP_009137117.1), ICP8 (YP_009137104), UL5 (YP_009137079.1), UL52 (YP_009137128), UL8 (YP_009137082). ICP8 is the *UL29* gene product. BBLF2/BBLF3 and ORF40/ORF41 are spliced transcripts.

function	Alpha		Beta		Gamma
	HSV-1/2	VZV	HCMV	HHV-6A/6B/7	EBV
DNA polymerase catalytic subunit	UL30	ORF28	UL54	U38	BALF5
DNA polymerase processivity subunit	UL42	ORF16	UL44	U27	BMRF1
single-strand DNA-binding protein	ICP8	ORF29	UL57	U41	BALF2
helicase/primase helicase subunit	UL5	ORF55	UL105	U77	BBLF4
helicase/primase primase subunit	UL52	ORF6	UL70	U43	BSLF1
helicase/primase subunit	UL8	ORF52	UL102	U74	BBLF2/BBLF3

HSV-1 is the most studied member of human herpesviruses. Initially, the functions of herpesvirus replication proteins were inferred from homology with HSV-1 proteins. In the last 20 years, the number of HSV-1 homologues that have been characterized in HCMV, EBV and KHSV has expanded. The homologues of UL30 [17–19], UL42 [19–22], ICP8 [23,24] and UL5/UL8/UL52 [25,26] have been purified, and their functions were confirmed to be aligned with that of HSV-1 proteins. However, HSV-1 remains one of the best characterized of the viral replication cycles. In this model replication system, six purified HSV-1 proteins can synthesize *in vitro* long concatemeric DNA products from a minicircular DNA substrate [27–29]. Despite these advances, precisely how the replisome assembles is not fully understood.

During infection, viral DNA is released from the capsid into the nucleus where viral DNA replication occurs [30]. Electron microscopy analysis reported that *in vivo* herpesvirus replication intermediates are linear concatemeric DNA molecules arranged in a head-to-tail fashion [31]. At later stages of infection, replication intermediates become highly branched DNA structures with X- and Y-junctions [32–36]. The first stage of HSV lytic replication was proposed to follow an origin-dependent replication (theta replication), involving the origin-binding protein UL9. However, theta-type replication DNA intermediates have not yet been observed either *in vivo* or *in vitro*. At least six replication proteins are recruited to the viral replication fork. The precise mechanism of replication remains controversial. Purified replication proteins can generate *in vitro* long concatemeric DNA products from a circular substrate [27–29]. As implied from *in vitro* data, the concatemeric replication intermediates could be formed by a rolling-circle replication. They could also be formed by a recombination-dependent replication [37–39].

**Figure 1.**
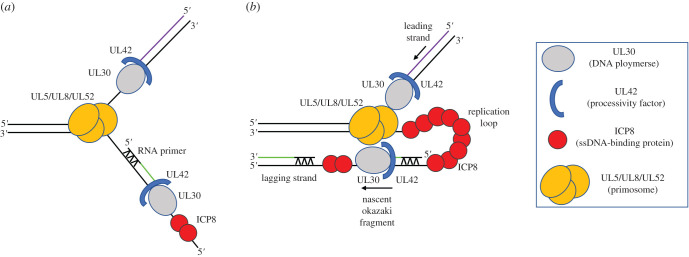
Model for the architecture of HSV-1 replisome. (*a*) HSV replication fork. (*b*) A replication loop is formed for coupled leading- and lagging-strand synthesis.

For an efficient viral DNA synthesis, the enzymatic reactions proceeding in opposite directions of the replication fork must be coordinated. Figure 1 presents a model for the architecture of the HSV-1 replisome for coordinated leading- and lagging-strand DNA synthesis. DNA synthesis is initiated by unwinding of duplex DNA by the UL5 helicase subunit of the UL5/UL8/UL52 complex. Single-stranded (ss) DNA binding by ICP8 prevents the reannealing of the separated strands. For replication to begin, herpes primase (UL52) must synthesize short RNA primers to provide a substrate for DNA polymerase. Then, DNA polymerase (UL30) can copy each strand. However, the configuration of HSV replication proteins at the replication fork is not known, as the atomic resolution structure of the replisome has not yet been determined. Thus far, the crystal structures of UL42 [40], ICP8 [41] and UL30 [42] have been solved. In particular, we lack a structural and molecular understanding of the helicase/primase complex of HSV-1 or any other herpesvirus. Here, we provide an overview of the state of knowledge of the ternary UL5/UL8/UL52 helicase primase complex. Insights from the molecular modelling of its subunits provide a detailed platform for discussion of the component functions, and prospects for targeted inhibition.

## 2. Structure and function relationships of subunits

The UL5/UL8/UL52 complex was initially identified by the observation of an enzyme with ATPase activity that is found specifically in HSV-1 infected Vero cells [43]. The partially purified approximately 440 kDa HSV-1 specific enzyme also exhibited GTPase and 5′–3′ helicase activity [43]. Characterization of the purified proteins revealed that the subunits were composed of three polypeptides with apparent molecular weights of 95 000 (UL5), 120 000 (UL52) and 70 000 (UL8) daltons [44] that associated with a 1 : 1 : 1 apparent stoichiometry [45]. Subsequent characterization of the complex revealed that it possesses single-stranded (ss) DNA-dependent NTPase, DNA helicase and DNA primase activities [44,46].

### 2.1. UL5 helicase subunit

**Figure 2.**
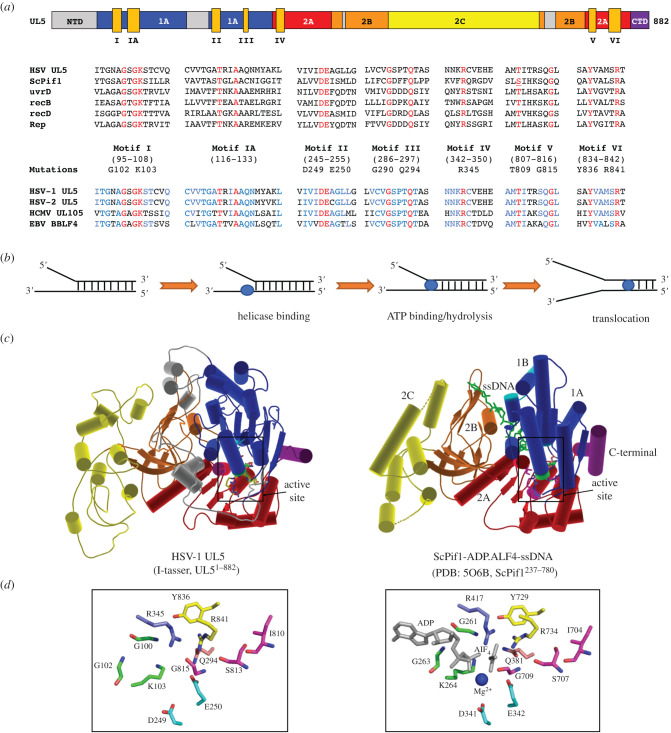
HSV-1 UL5 belongs to SF1 helicase superfamily. (*a*) Alignment of seven conserved motifs of HSV-1 UL5 (YP_009137079.1) with five members of SF1B superfamily: ScPif1 (NP_013650.1), UvrD (NP_418258.1), RecB (NP_417297.1), RecD (NP_417296.1), Rep (YP_026251.1), and with a presentative of each subfamily of herpesviridae: *Alpha* (HSV-2; YP_009137156.1), *Beta* (HCMV; YP_081551.1) and *Gammaherpesvirinae* (EBV; YP_401681.1). The residues conserved among all proteins are indicated in red; the ones conserved only within herpesviruses in blue. Helicase motifs of UL5: motif I (95–108), IA (116–133), II (245–255), III (286–297), IV (342–350), V (807–816), VI (834–842). (*b*) Schematic steps for helicase catalysis. (*c*) I-Tasser modelling [47–49] of UL5 based on the ternary complex of truncated ScPif1237–780 with ssDNA and ADP.ALF4 (PDB:5O6B) [50]. UL5-specific regions are shown in grey, ssDNA is shown in green. (*d*) Close-up view of conserved residues from each motif forming active site of helicase.

UL5 possesses seven motifs that are well-conserved in superfamily 1 (SF1) helicases including *E. coli* Rep, T4 bacteriophage Dda, eukaryotic Pif1 and SARS coronavirus ns13 (figure 2*a*) [51–53]. Helicases use the energy provided by DNA-stimulated NTP hydrolysis to achieve unwinding of duplex DNA. Thus, the catalytic reaction requires the coupling of the concerted events involving DNA binding, NTP binding, NTP hydrolysis and protein translocation along DNA (figure 2*b*). Consistent with a critical role for UL5 in the viral life cycle, mutations of the conserved UL5 helicase motifs (figure 2*a*) abolished the helicase activity of the complex [54,55] as well as viral replication *in vivo* [54]. Site-directed mutagenesis studies dissected the function of each motif during the pathway [55]. Residues in motif I (G102, K103) are directly involved in ssDNA stimulated NTP hydrolysis. Motif II (D249, E250) is required for coupling of DNA binding to ATP hydrolysis. The mutations of the conserved residues in motif III (G290, Q294), motif IV (R345), motif V (T809, G815) and motif VI (Y836, R841) compromise ATPase activity, and also undermine helicase activity suggesting these motifs are involved in the coupling of DNA binding and ATP hydrolysis to the process of DNA unwinding. SF1 motifs IA and III have been proposed to determine the translocation polarity of helicases [56]. UL5 translocates on and unwinds the duplex DNA in a 5′–3′ direction [43], and it can use ATP or GTP as an energy source [43,46].

To better understand UL5 structure, we generated a homology model of UL5 using I-Tasser [47–49]. Figure 2*c* shows the model of UL5 based on the structure of the prototypical member of Pif1 family helicases, *Saccharomyces cerevisiae* (Sc) Pif1 protein (PDB: 5O6B) [50]. Similar to other SF1 helicases, ScPif1 has a 4-domain architecture composed of the 1A, 1B, 2A and 2B domains [57]. The seven conserved helicase motifs (figure 2*a*) map to the cleft separating domains 1A and 2A (figure 2*c*). In the crystal structure of ScPif1, DNA is bound by residues from the 1A, 2A and 2B domains [50]. ScPif1 displays a yeast-specific domain (2C), which protrudes from domain 2B [50]. The role of this insertion domain, which is not present in the crystal structures of the bacteriodes [58] and mammalian [59] Pif1 helicases, is not known. It has been suggested that domain 2C may coordinate the helicase function through protein–protein interactions in cells [50]. Interestingly, HSV-1 UL5 also seems to display a similar insertion (grey, figure 2*c*, left). Figure 2*d* shows the close-up view of the helicase core formed by conserved residues from each motif. The N-and C-terminal regions are the least conserved regions among SF1 helicases, which is also the case for UL5. The terminal regions of SF1 helicases determine the substrate specificity of helicases and/or mediate protein–protein interactions [56].

The heterodimer of UL5 and UL52 binds to short single-stranded DNA oligonucleotides, with a minimum length of 12 nucleotides to activate ATPase activity [60]. While the Herpes helicase prefers to bind forked DNA substrates and ssDNA/dsDNA junctions, herpes primase interacts with the ssDNA tail, as determined by photo-cross-linking experiments [61]. In addition, unwinding of duplex DNA in a 5′ to 3′ direction requires a 5′ single-stranded overhang of greater than 6 nucleotides [62]. The rate of unwinding was found to be 2 bp s^−1^ [45], which is 25-fold lower than the estimated rate required for fork progression *in vivo* (50 bp s^−1^) [63]. Nonetheless, this rate can be stimulated *in vitro* by ICP8 up to 60 bp s^−1^ [64], as well as by UL8 [65,66], and by UL42 [67].

### 2.2. UL52 primase subunit

**Figure 3.**
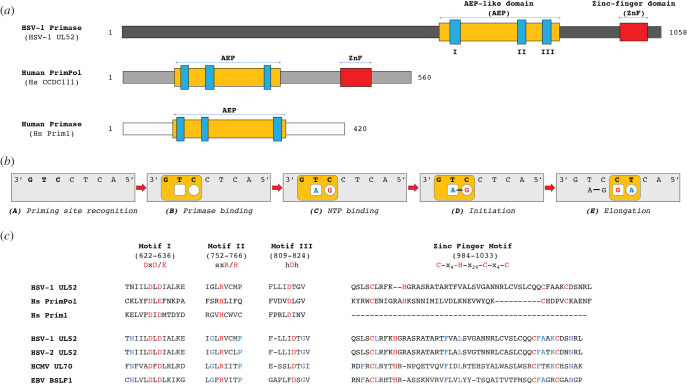
HSV-1 UL52 belongs to AEP primases. (*a*) Domain organization of a representative member of AEP superfamily: HSV-1 UL52 (NCLDV-herpesvirus primase clade), human PrimPol (Prim-Pol clade) and HsPrim1 (AEP proper clade). The catalytic motifs within AEP-like domain are shown in blue. (*b*) Multiple sequence alignments of HSV-1 UL52 with human PrimPol, human Prim1, and with a presentative of each subfamily of Herpesviridae. UL52 contains a conserved AEP domain (motif A, B and C), and a zinc finger domain. The residues conserved among all proteins are indicated in red; the ones conserved only within herpesviruses in blue. *x*, any residue; *h*, hydrophobic residue. NCBI's RefSeq accession numbers: HSV-1 UL52 (YP_009137128.1), HsCCDC111 (NP_689896.1), Hs Prim1 (NP_000937.1), HSV-2 UL52 (YP_009137205.1), HCMV UL70 (YP_081518.1) and EBV BSLF1 (YP_401662.1). (*c*) Hypothetical mechanism of primase synthesis.

Primases can be divided into two superfamilies: DnaG-like and archaea-eukaryotic primase (AEP)-like primases [68,69]. HSV-1 primase belongs to the AEP superfamily that includes the AEP proper clade, the NCLDV-herpesvirus clade and the PrimPol proper clade [69,70] (figure 3*a*). All members of the AEP superfamily contain a catalytic core (AEP-like domain), which is distinct from the topoisomerase-primase (TOPRIM) fold of the bacterial DnaG superfamily [68,69]. AEP-like domains have three signature motifs: motif I (DhE where *h* is a hydrophobic residue), motif II (sxH where *s* is a small residue, *x* could be any residue) and motif III (hDh) (figure 3*b*), all of which are conserved in herpes primase (figure 3*b*). Mutational analysis of motif I determined that D628 and D630 are critical for primase activity (figure 3*b*) [71,72]. Similar to other AEP primases, it is anticipated that the aspartate from motif III (D811) along with those from motif I (D628 and D630) will coordinate the catalytic divalent metals, while the arginine (R762) in motif II could interact with the incoming nucleotide during DNA polymerization [73].

UL52 also harbours a putative zinc finger domain (figure 3*a*) that is conserved in bacterial and bacteriophage primases, as well as human PrimPol (figure 3*a*) [74–77]. The putative UL52 zinc finger domain, Cys-X4-His-X29-Cys-X4-Cys, maps to residues 984–1033 (figure 3*b*) and mutation of the last two cysteines (C1023A and C1028A) significantly reduced DNA binding of the UL5/UL52 subcomplex as well as UL5/UL8/UL52 complex, and completely ablated its primase activity [78,79]. Furthermore, zinc finger mutants failed to support viral replication *in vivo* [79] underscoring a key role for this domain in mediating DNA binding, primase activity and viral replication.

In order to synthesize oligoribonucleotides on ssDNA, primases must first bind a DNA template, then their NTP substrates, and catalysis is initiated with the formation of a dinucleotide and inorganic pyrophosphate from two bound NTPs [80]. Additional NTPs are then incorporated at the 3′-end of the primer to generate full-length primers. Figure 3*b* depicts a general mechanism for primer synthesis. Herpes primase also follows these basic steps. UL52 can bind pyrimidine-rich DNA templates, such as poly-dT, and synthesize 2–4 nucleotides [44,46]. However, for synthesis of RNA primers longer than 4 nucleotides, UL52 requires a specific tri-nucleotide motif on ssDNA template: 3′-deoxyguanylate-pyrimidine-pyrimidine-5′ (3′-G-pyr-pyr- 5′) [81–83]. Similar to other primases, the zinc-binding domain on the C-terminus of UL52 could play a role in the recognition of the primase initiation site [75,84,85]. The 3′ deoxyguanylate of the recognition motif is required to position the primase at the initiation site, but not for the initiation of phosphodiester bond formation [86]. Once the di-ribonucleotide primers are synthesized (the rate-limiting step), the efficiency of their elongation is largely dependent on the length and sequence of template flanking regions [81,82]. Even when optimal DNA templates are provided, UL52 polymerizes primers of up to only 13 nucleotides, with the majority of products being limited to 2–3 nucleotides-long [82]. By contrast, the HSV-1 DNA polymerase (UL30) can elongate RNA primers to at least 8-nucleotides long [87]. Even so, the primase-coupled polymerase reaction is very inefficient, such that less than 2% of the primase-synthesized primers can be elongated [87]. Thus overall, UL52 is a relatively poor DNA primase. UL52 is not an accurate RNA polymerase either, with an average rate of misincorporation of one error per 30 NTPs synthesized during elongation [88]. Indeed, low fidelity is not a unique feature of herpes primase, but rather common among eukaryotic primases [86]. Fortunately, their high error rate does not reflect on the genome stability, since they are removed and replaced by DNAs. However, in the case of HSV-1, the low fidelity of UL52 could impact the primase-coupled polymerase activity as UL30 poorly elongates substrates containing mismatches at the 3′-terminus [89].

### 2.3. UL8 subunit

The UL8 subunit of the heterotrimeric complex does not possess any known intrinsic catalytic activity [90,91] yet, its biological function is strictly required for the *in vivo* viral genome synthesis [14–16,92]. As a component of the heterotrimeric UL8–UL52–UL5 complex, UL8 is critical for the optimal activity of the primase [93–95], and the helicase [65,66]. Besides the requirement for the UL8 gene product in DNA synthesis, it also plays a biological role in facilitating the transport of UL5 and UL52 into the nucleus [96–98]. Unless all three subunits are co-expressed, they are mostly cytoplasmic as examined by immunofluorescence and do not localize to the cell nucleus [96,98]. Biochemical analyses of UL8 DNA binding showed that UL8 does not bind oligonucleotides smaller than 50 nucleotides [99], but it can bind to 90-nucleotide-long oligonucleotides [100]. Intriguingly, UL8 does stably associate with long ssM13 DNA, and forms protein-ssDNA filamentous structures [100]. The nucleoprotein filament formation is a property shared by many ssDNA-binding proteins involved in recombination including archaeal RadA [101], bacterial RecA [102], HSV-1 ICP8 [103], KHSV ORF6 [24] and human Rad51 [104]. Compared to these proteins, UL8 filaments are distinct in that they are not helical filaments [100]. The significance of UL8 nucleofilament formation in an infected cell is currently unknown.

**Figure 4.**
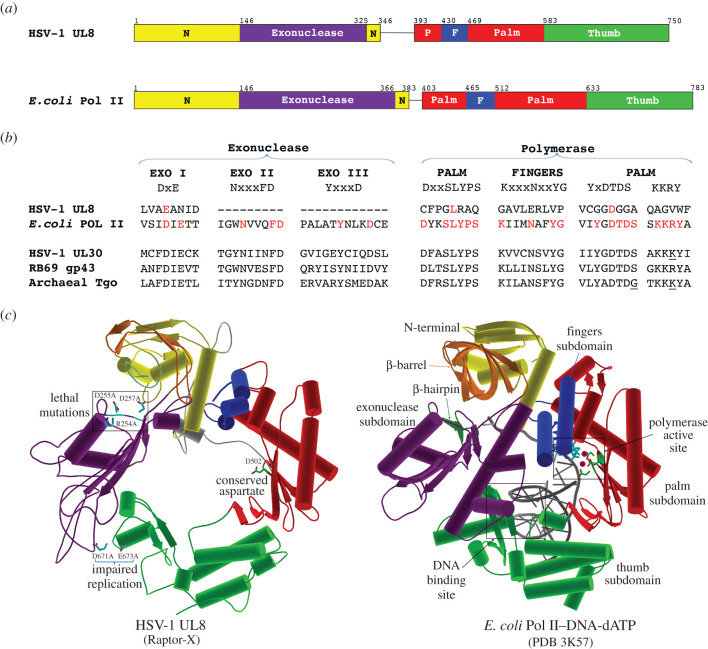
Domain organization of HSV-1 UL8 and *E. coli* Pol II. (*a*) Linear representation of amino acid sequences is adapted from reference [108]. (*b*) Multiple sequence alignments of HSV-1 UL8 (YP_009137082.1), *E. coli* Pol II (OSM09271.1), HSV-1 UL30 (YP_009137105.1), RB69 gp43 (NP_861746.1) and archaeal Tgo (ALL53335.1). (*c*) The structure of UL8 (left) was constructed using RaptorX software [107] based on the crystal structure of the ternary Pol II–DNA–dATP complex [108] (right). Right: template DNA is shown in grey, and dATP in cyan. The N-terminal domain contains N-terminal subdomain (yellow) and exonuclease subdomain (purple). β-barrel (orange) and β-hairpin (dark green) are located at N-terminal subdomain. Polymerase domain is composed of palm (red), fingers (blue) and thumb subdomains. Left: viruses carrying alanine substitution of residues R254, D255 and D257 caused complete inhibition, mutations of residues D671 and E673 caused impaired replication *in vivo* [109].

The C-terminal portion of UL8 was reported to be homologous to replicative B-family DNA polymerases [105], comprising the canonical three subdomains: fingers, palm and thumb [106]. Molecular modelling using Raptor X [107] of the full protein using the crystal structure of the ternary *E. coli* Pol II–DNA–dATP complex (PDB:3K57) [108] as a template structure suggests the polymerase fold is retained in UL8, but with significant divergence in its common polymerase motifs (figure 4). Out of three conserved catalytic aspartates located in the palm subdomain, DXXSLYPS from motif A and DTDS from motif C [110], only the first aspartate D502 from motif C (DGGA) is conserved in UL8 [105] (figure 4*b*). The palm subdomain of B-family polymerases also contains a conserved DNA-binding KKRY motif [111] that is absent in UL8 (figure 4*b*). The thumb subdomain in UL8 appears to also be divergent. However, the C-terminal 33 amino acids of UL8 (amino acids 717–750) are indispensable for HSV-1 replication [98] and expression plasmids encoding proteins carrying D671A and E673A mutations from the C-terminus displayed a replication-deficient phenotype in transient transfection experiments [109].

The N-terminus of UL8 is also significantly diverged from the exonuclease domain of B-family DNA polymerases (figure 4*a*). Three exonuclease motifs (Exo I, II and III) essential to perform the 3′–5′ exonuclease cleavage [112] are not conserved or are apparently absent in UL8 (figure 4*b*). N-terminal subdomains display the most structural diversity among polymerases [113], and dictate their specialized functions, such as translesion synthesis of Pol II [108] or RNAse H activity by UL30 [114]. On the other hand, the UL8 N-terminus carries residues essential for *in vivo* viral replication. For instance, deletion of 26 residues from the N-terminus (1–26) abolished DNA replication in transient transfection assays [98]. Another study reported that combined mutations of N-terminal R254, D255 and D257 residues caused a replication-deficient phenotype [109]. Thus, both N- and C-terminal regions appear to be indispensable for replication. Overall, divergence of UL8 polymerase motifs suggests this subunit may play a DNA binding and structural scaffolding role as a component of the helicase–primase complex, rather than a direct catalytic role. More work is required to delineate the precise molecular functions of UL8.

## 3. Interactions between subunits

**Figure 5.**
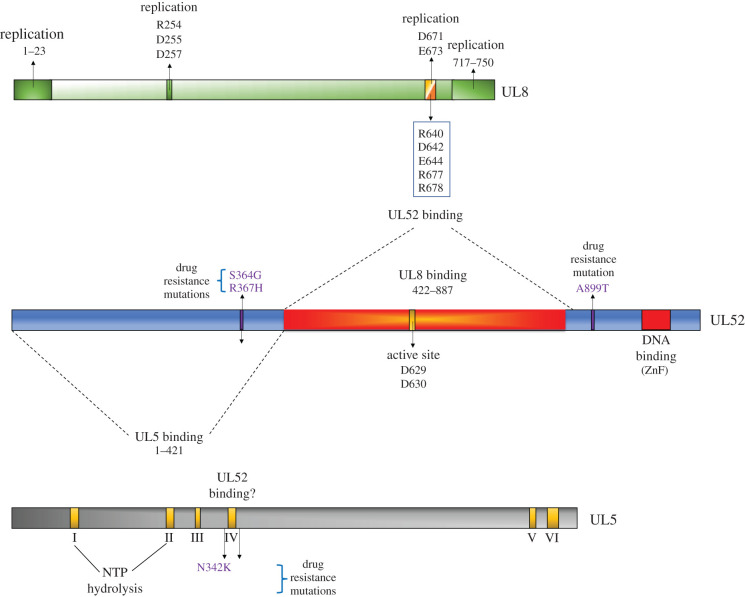
Map of interactions between subunits.

UL5, UL52 and UL8 together form a stable complex even in the presence of high salt (500 mM) [115]. All possible combinations of two-subunit heterodimers (UL5/UL52, UL5/UL8 and UL52/UL8) also form a stable complex and can be co-purified suggesting that each subunit interacts with each other. The basis for interactions between subunits has been largely explored [109,116,117]. Mutagenesis experiments identified a triple mutant (R640A–D642A–E644A), and a double mutant (R677A–R678A) of UL8 that lost the ability to bind UL52, suggesting that these residues interact with the UL52 subunit [109]. Further, the middle domain of UL52 (residues 422–887) interacts with the residues from the C-terminus of UL8 [117] (figure 5). The C-terminus of UL8 (718–750, minimally residues 727–739) also interacts with UL30 [118]. Additional observations reveal that UL8 interacts with both primase and polymerase via its C-terminal thumb-like subdomain (aa 583–750). Taken together, these data suggest that the UL8 C-terminus serves as a bridge to align the catalytic sites of primase and polymerase.

The UL52 N-terminus (aa 367–421) interacts with UL5 [117]. Less is known about how UL5 interacts with the other subunits, however, mutations in UL5 motif I (G102, K103) and motif IV (R345) eliminated helicase activity, but they did not abolish UL5 binding with either UL52 or UL8 [54]. The less conserved N- and C-terminal domains on UL5 could also be potential binding sites, as suggested for other helicases [56]. As will be discussed in §5 (below), a class of anti-herpes drugs targeting helicase and primase act by interacting with both UL5 and UL52. Interestingly, mutations which conferred resistance against helicase/primase inhibitors (HPIs), are observed on both UL5 and UL52 [119]. Thus, these mutated residues possibly map to the UL5–UL52 interface (see below). Three resistance mutations were found in UL52. Two of them were located on the UL52 N-terminus, residues S364 and R367, which happen to be within the region shown to be essential for UL5 binding [117]. Indeed, UL52 mutants S364G and R367H were defective in UL5–UL52 complex formation [117]. The third resistance mutation A899 maps to the UL52 C-terminus [119]. The C-terminal mutant A899T did not affect the UL5–UL52 interaction [117]. Resistance mutants in UL5 (G352 V, M355I and K356T) are all downstream of motif I [119]. However, none of the UL5 variants had an effect on UL52 binding [117]. More in-depth analysis is required to explore protein binding sites on UL5.

## 4. Functional interdependence between subunits

A fully functional helicase/primase complex requires protein–protein interactions between UL5, UL8 and UL52.

### 4.1. UL5/UL8

In isolation, a UL5/UL8 binary complex does not exhibit DNA-dependent ATPase activity [95]. This observation might reflect the inability of UL5/UL8 to bind DNA substrates used in the helicase assay (dsDNA flanked by ssDNA regions). However, cross-linking assays showed that UL5/UL8 can still bind this substrate [95]. The pattern of cross-linking products formed with UL5/UL8 was different than the DNA–protein complex formed with UL5/UL8/UL52, which suggests that UL52 could modulate the way UL5 binds DNA and stimulates DNA-dependent ATPase activity of the complex [95].

### 4.2. UL52/UL8

Similar to UL5/UL8, a UL52/UL8 binary complex is not fully functional in that it does not synthesize RNA primers *de novo* on ssDNA template. This deficit is corroborated by cross-linking experiments suggesting that that UL52/UL8 does not bind the primase substrate used [95]. Interestingly, UL52/UL8 does synthesize NTPs on an RNA primer-DNA template duplex that lacks the requirement for the primase initiation step. Thus, UL52/UL8 acts as a functional RNA polymerase, and the minimal complex can form phosphodiester bond without UL5. The elongation of an RNA primer-DNA template by the UL52/UL8 complex is kinetically less efficient than that of the UL5/UL8/UL52 complex. Even though *k*_cat_ of the reaction was slightly decreased (8.1 h^−1^ versus 2.3 h^−1^), the catalytic efficiency (*k*_cat_/*K*_M_) of the subcomplex was approximately 20-fold decreased due to the increased *K*_M_ for NTPs [95]. In summary, UL5 is required for the initiation of RNA primer synthesis, and maximal primase activity of the UL52/UL8/UL5 ternary complex.

### 4.3. UL5/UL52

The UL5 helicase and UL52 primase subunits are also interdependent. Unless these proteins are co-translated in the cells, they are apparently inactive [46,90], suggesting that the UL5/UL52 inter-subunit interactions are important for their proper folding, substrate interactions and/or activity. Mutational analysis of both subunits revealed that their interdependence is complex. Inactivation of the primase activity of UL52, by targeting the structure of the zinc-binding domain (C1023A and C1028A), also inactivates the helicase activity [78]. However, mutations targeting primase active site (D628 and D630), do not impair helicase activity [71,72]. Strikingly, inhibiting the helicase activity of UL5 by mutating the conserved helicase motifs (motifs I–VI; figure 2*a*) increases the primase activity up to 36-fold [55]. The basis for UL5/UL52 functional interdependence merits further investigation. It has been suggested that their mutual dependence may be due to their shared DNA interaction sites. The DNA-binding footprint of UL5 in the absence of UL52 is substantially different than that of the UL5/UL52 complex [95]. Hence, UL52 may impart structural rearrangements or expand the UL5 DNA–protein interface.

In conclusion, UL5 and UL52 display a complex relationship. The interdependence of helicase and primase activities, common in all DNA replication systems, is likely to be requisite to regulate the two activities at the replication fork, as the two activities must proceed in opposite directions along the same DNA strand. The molecular underpinnings of helicase/primase coupling in different replication systems requires more study.

### 4.4. UL5/UL52/UL8

Standard biochemical assays show that the subassembly of UL5/UL52 can synthesize primers on homopolymer DNA substrates of low sequence complexity (poly-dT) or unwind short duplex DNA as efficiently as the UL5/UL8/UL52 complex. However, in the absence of UL8, UL5/UL52 is not sufficient for *in vivo* DNA replication, primer synthesis on more complex DNA substrates (ssM13) [93], primase-coupled polymerase activity [87,93], lagging-strand synthesis [27,93] or unwinding of duplex DNA longer than 30 nucleotides [66]. Thus, HSV-1 DNA replication can occur only when all three subunits collaborate together.

## 5. Concluding remarks and outlook—helicase and primase as drug targets

Nucleoside analogues that target virus-encoded polymerase have dominated the anti-herpes drug industry for decades. The first nucleoside analogue drug, acyclovir, was discovered in 1977 by Elion *et al.* [120]. The first non-nucleoside analogue targeting helicase/primase complex (T157602) was reported in 1998 [121]. Since then, two HPIs have emerged as promising drug candidates: pritelivir (BAY 57–1293) [122] and amenamevir (ASP2151) [123]. Both drugs were shown to be more effective than acyclovir in animal models [123–126]. BAY 57–1293 successfully completed a phase II clinical trial for treatment of HSV-2 in 2016 [127,128]. As of 2021, the medicine has entered a phase III clinical trial for immunocompetent patients who have acyclovir-resistant mucocutaneous HSV infections. However, due to safety concerns, the clinical trial of ASP2151 was discontinued at phase I in 2014, in the US [129]. Interestingly, Amenamevir successfully completed a randomized phase III clinical study in Japan, in 2017 [130]. Amenamevir is currently used for the treatment of shingles (herpes zoster) and well tolerated in Japanese patients.

The efficacy of HPIs has been confounded by rapid development of viral resistance to these agents. Interestingly, resistance mutations were pre-existing in HSV-1 clinical isolates that had not been selected by the drugs [131]. The majority of variants found in strains resistant to BAY 57–1293 map to residues C-terminal of the conserved helicase motif IV (G352C, G352R, M355T, K356N, K356T, K356Q), with one mutation (N342 K) found within motif IV of UL5 [119] (figure 5). On the other hand, a single mutant virus (A899T) was found in the UL52 gene [132], suggesting that the mode of action of BAY 57–1293 is mainly via UL5. Nevertheless, a virus strain harbouring a K356T mutation on UL5 and an A899T mutation on UL52, conferred 2500-fold resistance to BAY 57–1293 [132]. Resistance mutations were also observed against ASP2151. The amino acid changes were G352 V and M355I in UL5; S364G and R367H on UL52 [133].

UL5 and UL52 are essential in the herpesvirus life cycle, and highly conserved among all members of herpesviridae. Therefore, compounds inhibiting the helicase/primase complex could be used as broad-spectrum drugs targeting all human herpesviruses. However, none of the current HPIs under development are effective against β- and γ-herpesvirinae [119]. In conclusion, the development of HPIs as an alternative treatment to the currently employed therapeutics requires more extensive work and will especially benefit from the resolution of the three-dimensional structure of the UL8-UL5-UL52 complex to facilitate rational drug design.

## Data accessibility

This article has no additional data.
